# The use of complementary and alternative medicine by adults with allergies: a Czech national representative survey

**DOI:** 10.1186/s12906-021-03316-z

**Published:** 2021-06-14

**Authors:** Jitka Pokladnikova, A-La Park, Jan Draessler, Anna Lukacisinova, Irena Krcmova

**Affiliations:** 1 Department of Social and Clinical Pharmacy, Faculty of Pharmacy, Charles University, Hradec Králové, Czech Republic; 2 Department of Health Policy, Personal Social Services Research Unit, The London School of Economics and Political Science, London, UK; 3 Department of Informatics and Quantitative Methods, Faculty of Informatics and Management, University of Hradec Kralove, Hradec Králové, Czech Republic; 4 Department of Clinical Immunology and Allergy, Charles University, University Hospital Hradec Kralove, Hradec Králové, Czech Republic

**Keywords:** Allergy, Complementary and alternative medicine, Czech Republic, Utilization, A cross-sectional survey

## Abstract

**Background:**

The prevalence rates of the use of Complementary and Alternative Medicine (CAM) in allergy patients range from 42% in the United States up to 50% in Europe. In the Czech Republic, no such data exists. Our aim was to examine patterns in CAM use in populations with self-reported allergies in the Czech Republic.

**Methods:**

A cross-sectional survey was conducted. A sample of citizens aged 15 years and older, sex, age, and region-stratified, was randomly selected from the 2014 voter registration lists (*n* = 8,395,132). Respondents with self-reported allergies were further analyzed.

**Results:**

Overall, 93% of the respondents with self-reported allergies reported the use of 1 or more CAM modalities during the past 30 days. Herbal teas, relaxation techniques, a detoxifying diet, dietary supplements (excluding vitamins and minerals), and reflexology were used in respondents with allergies. Females, under age 30, with higher education, higher income, and self-reported poor health, were significantly associated with the use of CAM among respondents with allergies.

**Conclusions:**

The prevalence of CAM use among people with self-reported allergies in the Czech Republic is higher compared to other countries, with determinants of CAM varying across specific CAM categories. More attention to existing use is needed to promote the healthy adoption of CAM by raising awareness of its safe and effective use, both for CAM users as well as for health care providers.

## Background

According to World Allergy Organization (WAO) statistics, worldwide hundreds of millions of people suffer from allergic rhinitis, and 300 million from asthma [1]. Allergies are the most common chronic disease in the Czech Republic as well as in Europe, affecting up to 24 and 20% of the population, respectively [1, 2]. The European Academy of Allergy and Clinical Immunology (EAACI) predicts that by 2025 half of the entire EU population will suffer from chronic allergic diseases [3]. The WAO as well as EAACI has issued a clarion call to policymakers to counter the growing global public health burden posed by allergies, and has highlighted the need to implement policy actions to address this challenge [3]. For the general population, a primary focus has been publicizing the benefits of Complementary and Alternative Medicine (CAM) therapies for the enhancement of overall wellness and wellbeing, rather than arguments focused on dissatisfaction with conventional medicine, or attempts to improve the effectiveness of conventional therapies by combining them with CAM [4, 5]. Nevertheless, patients with allergies most commonly use CAM out of fear over conventional therapies, their belief that CAM is safer than conventional therapy options, as well as a wish to try alternatives following unsatisfactory results from conventional therapies [6, 7]. The prevalence rates of CAM use in allergy patients range from 42% in the United States between 30 and 50% in Europe [8–10]. Thus CAM use in the population with allergies is extensive. Types of CAM use are diverse, with the most commonly reported methods being herbal medicines, acupuncture, or homeopathy [10]. Although CAM users among patients with allergies are often defined as being female, younger to middle-age, and with higher education, little information exists on the characteristics of CAM users by types of allergies or types of CAM modalities [10].

Therefore, we aim to establish the prevalence rates of CAM use among respondents with allergies in a nationally representative sample in the Czech Republic and to identify patterns of CAM use including predictors for overall and sub-categories of CAM use and reasons for CAM use.

## Methods

### Study design

A cross-sectional survey was conducted from November to December 2014. It used face-to-face interviews to get information from the general population aged 15 and over on the use of CAM by residents of the Czech Republic [11]. A secondary analysis focuses on allergy self-reports.

### Respondents

Random quota sampling was used to select a sample population from voter registration lists (*n* = 8,395,132). Assuming a confidence level of 99% with a margin of error of 3%, the sample size was calculated to be 1810 respondents. The aim was to address a cohort aged 15 years and over which would be representative of the population of the Czech Republic in terms of sex, age, and regional distribution. Of 14,777 electoral districts in the country, 180 were randomly selected. Trained interviewers contacted individuals from randomly selected streets and houses in each of these electoral districts to conduct face-to-face interviews.

### Data source

Face-to-face interviews were conducted between November and December 2014 to gather data on demographics, self-reported health, CAM use, and reasons for CAM use. A list of 29 conditions together with an “other” option as an open-ended option was provided to respondents to help them self-report chronic health conditions. Those with allergies were instructed to specify the allergy type. Self-reported health was rated on a five-point Likert scale ranging from “excellent” =1, “very good” = 2, “good” = 3, “poor” = 4, “very poor” = 5, and negative feelings, if any, were reported using the yes/no option. A list of CAM modalities was created, including less conventional practices identified based on the assessment of the population’s current knowledge regarding CAM use. Respondents were instructed to choose from the list the CAM modalities used by them during the last 30 days. The listed items included vitamins and minerals, herbal teas, aromatherapy, homeopathy, Bach flower remedies, gemmotherapy, non-vitamin/non-mineral dietary supplements, special diets, detoxification, chiropractic, massage, reflexology, yoga, relaxation, visual imagery, biofeedback, hypnosis, Ayurveda, Traditional Chinese Medicine (TCM) (such as acupuncture, Chinese herbs, and herbal medicines), energy healing, ethicotherapy, meditation, and prayer. The responses to open-ended questions about the use of unlisted CAM modalities and reasons for it allowed further classification of CAM therapies into five categories as recommended by the National Centre for Complementary and Integrative Health (NCCIH) [12].

The final draft of the questionnaire was pretested on a convenience population sample of different sex, age, and education (*N* = 213). Details on the standardization and administration of interviews as well as the development of the survey have previously been published [11].

The data were collected by Inres –Sones, v.o.s. (Prague, Czech Republic), a private agency providing services in non-commercial sociological and socio-psychological research to public institutions, research institutes, universities, and foundations since 1993 [13]. The study participants were volunteers who received no financial incentive. The only exclusion criterion was cognitive inability to complete interviews. As this was an anonymous survey and presented minimal risk of harm to human participants, an implied oral informed consent was obtained prior to commencing the interview. The study was approved by the Ethical Committee of the Faculty of Pharmacy.

### Statistical analysis

Descriptive statistics were provided for variables of interest. To determine predictors of CAM use in people with self-reported allergies, a binary regression was conducted with data balancing so that the ratio of the two groups was approximately 1:1. To construct the regression model, we used the forward-selection method, keeping significant predictors based on the Wald statistics. Dependent variables were CAM modalities and overall CAM use, and independent variables were sex, categorized age, education, monthly household income, marital status, religious affiliation status, the experience of negative feelings, place of residence category, and self-rated health category. In individual models, for selected variables, some of the categories were merged where there were a small number of respondents (household income, self-rated health, size of the place of residence, and age). Employment status was omitted due to insufficient sample size, as only four respondents with allergies reported being unemployed. The significance level was 0.05. Statistical analysis was performed using IBM SPSS version 24 [14]. The data quality was ensured through conversion to an electronic format by trained staff using the SASD 1.4.10 software with built-in control functions [13]. Data from 200 questionnaire copies, i.e., 11%, were entered twice by two independent operators to check coding errors.

## Results

In total, 2204 people were initially considered in our present study. Of these, 394 (17.9%) declined to participate in the survey because of lack of time (48.6%) or distrust or lack of interest (22.8%). The final sample of 1810 respondents consisted of 187 (10.3%) with self-reported allergies. The socio-demographic characteristics of the respondents are shown in Table 1.

**Table 1:** Descriptive characteristics of respondents with self-reported allergy (*N* = 187)

Sex, n (%)	
Male	84 (44.9)
Female	103 (55.1)
Age, mean, n (%)	37.2
15–29 years	82 (43.9)
30–39 years	30 (16.0)
40–59 years	53 (28.3)
60 years and more	22 (11.8)
Education, n (%)	
Less than high school	16 (8.6)
High school	131 (70.1)
University	40 (21.4)
Mohthly household income in CZK, n (%)	
< = 1000	9 (4.8)
10,001–20,000	40 (21.4)
20,001–40,000	100 (53.5)
> 40,000	38 (20.3)
Martial status, n (%)	
Living together	106 (56.7)
Single/separated/ divorced/widowed	81 (43.3)
Negative feelings, n (%)	132 (70.6)
Self-rated health, n (%)	
Excellent	46 (24.6)
Very good	99 (52.9)
Good	36 (19.3)
Poor and very poor	6 (3.2)
Place of residence, n (%)	
1–499 inhabitants	20 (10.7)
500–1999 inhabitants	37 (19.8)
2000–4999 inhabitants	35 (18.7)
5000–19,999 inhabitants	33 (17.6)
20,000–99,999 inhabitants	23 (12.3)
100,000 inhabitants and more	39 (20.9)
Unemployed, n (%)	4 (2.1)
Type of allergy, n (%)	
Allergic rhitinis	74 (39.6)
Asthma bronchiale	37 (19.8)
Skin allergy	34 (18.2)
Food allergy	7 (3.7)
Allergy non-specified	36 (19.3)
Other health conditions^a^, n (%)	
High blood pressure	26 (13.9)
Musculoskeletal disorders	26 (13.9)
Otorhinolaryngology problems	19 (10.2)

^a^Health conditions of prevalence more than 10% are listed

CZK, Czech Crowns (EUR = 27.533 CZK (2014))

### CAM use by types of therapies

One hundred and seventy-three (92.5%) respondents with self-reported allergies reported the use of some kinds of CAM within the past 30 days. Biologically based therapies were the main drivers for any CAM use (85.0%), followed by the mind (33.7%), and body-based therapies (29.4%). Herbal teas (64.2%), relaxation techniques (25.1%), detoxifying diet (6.4%), dietary supplements (excluding vitamins and minerals) (5.3%), and reflexology (4.3%) were used in respondents with allergies. The numbers of respondents using a CAM therapy are shown in Table 2.

**Table 2:** Use of complementary and alternative medicine in the past 30 days by type of therapy in respondents with self-reported allergy (*N* = 187)

	N (%)
Using CAM	173 (92.5)
Bio CAM	159 (85.0)
Herbal teas	120 (64.2)
Vitamins & minerals	113 (60.4)
Detoxification	12 (6.4)
Dietary supplements excluding vitamins & minerals	10 (5.3)
Special diet	9 (4.8)
Aromatherapy	6 (3.2)
Gemmotherapy	1 (0.5)
Mind-body CAM	63 (33.7)
Relaxation	47 (25.1)
Yoga	18 (9.6)
Meditation	5 (2.7)
Hypnosis	1 (0.5)
Psychotherapies	1 (0.5)
Visual imagery	0 (0.0)
Biofeedback	0 (0.0)
Others	10 (5.3)
Body-based CAM	55 (29.4)
Massage	55 (29.4)
Reflexology	8 (4.3)
Chiropractic	1 (0.5)
Whole system therapies	10 (5.3)
Homeopathy	9 (4.8)
Ayurveda	1 (0.5)
Chinese medicine	2 (1.1)
Energy CAM	3 (1.6)
Energy healing	2 (1.1)
Others	1 (0.5)

### CAM use by types of allergic conditions

For people experiencing allergic rhinitis, herbal teas (67.6%) were most-commonly used, followed by vitamins and minerals (54.1%), relaxation techniques (25.7%), and massages (23.0%). On the other hand, for people with asthma, vitamins and minerals (73.0%) dominated, followed by herbal teas (59.5%), massages (35.1%), and relaxation techniques (21.6%). For people with skin allergies, herbal teas (67.6%) were the most popular, ahead of vitamins and minerals (61.8%), massage (29.4%), relaxation techniques (29.4%), and yoga (17.6%).

### Predictors of CAM use in allergy respondents

Predictors of overall CAM use among respondents with allergies were being female, under the age of 30, higher education, higher income and self-reported poor health (Table 3).

**Table 3:** Predictors of Complementary and Alternative Medicine Use by type of CAM category among respondents with self-reported allergy (*N* = 187)

	Use CAM		Biologically CAM		Body-based CAM		Whole system CAM		Mind-body CAM	
	PV	OR (95% CI)	PV	OR (95% CI)	PV	OR (95% CI)	PV	OR (95% CI)	PV	OR (95% CI)
Sex (Male)	0.005**	0.357 (0.17, 0.74)	0.002**	0.386 (0.21, 0.70)						
Monthly household income in CZK	0.026*						< 0.001**		0.044*	
< 20,000	Ref						Ref		Ref	
20,001–40,000	NS	2.039 (0.88, 4.70)					0.002**	0.121 (0.03, 0.47)	NS	1.335 (0.70, 2.56)
> 40,000	0.007**	4.992 (1.55, 16.08)					0.027*	4.377 (1.18, 16.19)	0.013*	3.032 (1.26, 7.31)
Self-rated health	< 0.001				0.004**		< 0.001**		0.029*	
Neutral - Very bad	Ref				Ref		Ref		Ref	
Good	< 0.001**	0.156 (0.06, 0.43)			0.011*	0.570 (0.37, 0.88)	< 0.001**	0.045 (0.01, 0.19)	NS	0.659 (0.35, 1.23)
Very good	NS	0.732 (0.22, 2.48)			0.034*	1.847 (1.05, 3.26)	NS	0.495 (0.12, 1.96)	NS	1.792 (0.82, 3.94)
Education	< 0.001**		0.012*				< 0.001**			
Less than high school	Ref		Ref				Ref			
High school	< 0.001**	19.702 (5.46, 71.13)	0.005**	2.065 (1.24, 3.44)			NS	0.906 (0.36, 2.27)		
University	NS	2.279 (0.65, 7.99)	NS	0.902 (0.48, 1.71)			< 0.001**	17.233 (3.58, 83.06)		
Religious identity (Yes)									0.005**	0.409 (0.22, 0.77)
Martial status										
Simple, separated, widowed, divorced							Ref			
Living together							0.003**	0.168 (0.05, 0.55)		
Age (years)	0.001**									
15–29	Ref									
30–39	0.019*	0.282 (0.10, 0.81)								
40 and over	< 0.001**	0.174 (0.07, 0.45)								
Place of residence (inhabitants)							0.002**			
1–1999							Ref			
2000–4999							NS	3.407 (0.87, 13.41)		
5000–19,999							NS	1.068 (0.20, 5.73)		
20,000–99,999							< 0.001**	27.475 (4.96, 152.30)		
100,000 and more							NS	3.610 (1.00, 13.04)		

* significant at 0.05

** significant at 0.01

*Ref* – reference category, *NS* – not significant, *PV* – *p*-value for Binary logistic regression, forward method with Wald test

For people with allergies, men on average had significantly lower use of biologically based CAM modalities (64%) than women (OR 0.39, 95% CI 0.21–0.70, *P* = 0.002), while high school graduates had higher use of biologically based CAM than those with less than high school education (OR 2.07, 95% CI 1.24–3.44, *p* = 0.005).

In the mind-body therapy category, the most significant predictor was the religious affiliation status: those affiliated with a religion had half the chance of CAM use (OR 0.41, 95% CI 0.22–0.77, *p* = 0.005). The second most significant predictor was income. The chance of using mind-body methods was three times as high in the category with incomes over CZK 40,000 (OR = 3.03, 95% CI 1.26–7.31, *p* = 0.013).

Respondents who rated their health very positively used body-based CAM modalities almost twice as high often (OR 1.85, 95% CI 1.05–3.26, *P* = 0.034).

Energy-based and traditional whole system therapies could not be analyzed due to the low number of respondents.

### Reasons for CAM use

Although preventive use was identified as the primary strategy for CAM use, curative purposes were reported more frequently (17.9%). Using CAM as a proactive approach to maintaining good health was found to be another significant factor for choosing a CAM therapy (9.2%). The reasons for CAM use are shown in Table 4.

**Table 4:** Reasons for CAM use (*N* = 173)

	N (%)
Disease prevention	112 (64.7)
Own decision	68 (39.3)
Disease treatment	31 (17.9)
Advice of family members/friends	25 (14.5)
Recommendation of a physician	23 (13.3)
Proactive approach to health	16 (9.2)
Out of curiosity	11 (6.4)
A holistic health philosophy	10 (5.8)
Information on the Internet	9 (5.2)
Preference for more natural and milder therapy modalities	8 (4.6)
Recommendation of a pharmacist	8 (4.6)
Attempt to improve the effectiveness of conventional therapy	5 (2.9)
Fear of drug side effects	5 (2.9)
Interest in spirituality or personal growth	5 (2.9)
Affordability	3 (1.7)
Dissatisfaction with the outcome of therapy with conventional medicine	2 (1.2)
Influence of advertising	2 (1.2)
Individually based/personal approach of the therapist	0 (0.0)
Others	3 (1.7)

## Discussion

Our study aimed to establish the rate of prevalence for CAM use among respondents experiencing allergies in a nationally representative sample and to identify predictors for overall and individual categories of CAM use, as well as reasons for CAM use.

In our study, the prevalence of CAM use among respondents with self-reported allergies was higher compared to respondents from other countries (93% vs range of 68 to 86%) [7–11, 15–19]. Herbal teas and vitamins/minerals were the top reported CAM therapies among respondents with allergic rhinitis/skin allergy and asthma. These findings correspond to results from other countries except for Germany where patients suffering from different skin disorders used mainly homeopathy, autologous blood injections, acupuncture, and bioresonance [6]. The high use of dietary supplements and herbal remedies in the Czech Republic when compared to other countries may be partially explained by the fact that they are more accessible and available to the general population (from pharmacies and supermarket chains) and comparatively low cost. Moreover, herbalism has a long family tradition in the Czech Republic, with most of the population receptive to the use of local medicinal plants. Although some promising evidence exists related to the effectiveness of herb preparations, acupuncture, homeopathy, or yoga in the treatment of allergies, concerns regarding CAM use raised by health care professionals center on the overall paucity of data on CAM effectiveness, coupled with the low methodological quality of studies [20–24].

Therefore, making recommendations for or against CAM use is difficult. Also, a lack of communication between patients/service users and health care providers as well as insufficient knowledge and professional training opportunities for CAM among health care professionals may lead to less optimal use of various CAM modalities [25, 26].

For allergies, this might, for example, imply poor control of asthma, the possible unforeseen interactions between conventional medications and herbal medicines, or potential adverse effects from CAM [27–30].

The results of international pharmacovigilance monitoring drug safety issues for licensed drugs for more than 40 years shows patterns of immediate allergic adverse reactions associated with herbal medicines and reports a large number of different herbal medicines as causing immediate allergy-like reactions in the population [31]. The two most frequently reported immediate allergy-like reactions were skin and anaphylactic/anaphylactoid reactions, both of which were most frequently observed after oral administration [31]. Relaxation techniques and massage as widely used CAM modalities in our study also give rise to safety issues including ineffectiveness for people at risk [32]. For example, progressive muscle relaxations may trigger asthma in patients with low magnesium levels. These techniques are usually delivered in office-based psychotherapies and home-based relaxation practices. In particular, the latter case merits more attention as progressive muscle relaxation techniques can also trigger anxiety and depression among predisposed patients [24, 29, 32, 33]. It is well-known that people with asthma tend to suffer from symptoms of anxiety and depression [34].

In our study, the overall use of CAMs within respondents with self-reported allergies was significantly associated with female gender, under the age of 30, high school or higher education, higher income, and self-reported poor health. Similar results, including symptom burdens, and financial obstacles to accessing care, were reported by studies in Europe and United States [10, 35]. The high cost of health care and prescription drugs for asthmatics in some countries may create incentives to use CAM in place of conventional medicine. As health care in the Czech Republic is universally available, the cost of professional health care and prescriptions for conventional medications may not factor into decisions about whether or not to purchase CAM therapies. The potential predictors for biologically based therapies were that the user was a woman with a higher education degree. A similar pattern was found in a study of adults with asthma in the US, where women with higher incomes and more comorbidities were reported as having predisposing characteristics for dietary supplement use [15, 36]. More developed rationale (through education), higher affordability (as a result of higher incomes), and a greater interest in a healthy lifestyle among educated women may play a role in choosing dietary supplements [37]. Being an atheist with high income was also associated with the use of mind-body therapies. Similarly, other studies report a higher prevalence of mind-body CAM use in higher income and religious/spiritual respondents [38, 39]. Statistics show that two-thirds of the Czech population identify as irreligious, yet 44% believe in the existence of the soul [40]. Although we collected data on religious identity, we did not measure spirituality as a sole social construct in this study. Thus, we may only hypothesize that non-religious people in our study may have turned to the use of mind-body medicine because of a congruency between their personal values and beliefs and the “New Age” associations of some mind-body CAM techniques, and/or the increased likelihood that ‘spiritual needs’ could be met through these types of practices. Body-based therapy use was positively associated with experiencing better self-rated overall health. Massage (as the most frequently reported therapy) is associated with reduced physical pain, muscle tension, stress, anxiety, and depression. Since allergy conditions, especially asthma, can lead to prolonged stress and associated tension and negative mood, massage can contribute both to the reduction of muscle tension, stress, elevated mood, and enhancement of overall health and wellbeing [24, 34, 41]. Differences in those findings may be associated with specific preferences in different populations driven by socio-demographic, cultural, and financial background, as well as by the methodology used within the investigation of the patients’ preferences.

A variety of reasons exist for the use of any type of CAM, ranging from desires to prevent/cure/treat a medical condition, patients’ preferences for natural means of treatment, dissatisfaction with the effect of traditional medicine and fear of their side effects, to ease of access and relative financial affordability for many CAM treatments [4, 5]. Aside from the desire to cure a disease, a proactive approach to maintaining good health was found to be the most cited reason for CAM use in our study.

One of the strengths of our present study is the high response rate and assessment of predictors per CAM category. Broader definitions for CAM modalities included minerals/vitamins and dietary supplements, although some may argue these are conventional therapies. Therefore, CAM use may have been overreported in our survey. However, less conventional CAM modalities were also included on the list to better reflect actual population practices in the Czech Republic. A broader definition makes it possible to compare overall CAM use between countries. One limitation is the cross-sectional study design, where associations can be determined, but causality cannot be verified. Other limitations include possible misclassification and recall bias due to self-reporting. Further, occasional CAM users may have been missed and thus CAM use may have been underreported because of the short recall period, unlike in the case with annual use or lifetime prevalence rates.

## Conclusions

The use of CAM modalities among respondents suffering from allergic conditions is higher than that of the general population in the Czech Republic, with determinants of CAM varying across specific therapies. More attention and study is needed to enable the promotion of CAM by raising awareness of its safe and effective use, depending on individual conditions in terms of types and severity of allergies, and expanding from current primary CAM users (highly educated, young women with self-reported poor health) to include healthy adoption by more vulnerable CAM users and by health care providers. Future research should explore the impacts of the use of CAM modalities on quality of life in people with allergic conditions. Given a lack of economic evidence in the CAM field, this information could be fed into economic evaluations, to aid decision-makers in determinations of how to allocate resources more efficiently among CAM modalities.

## Data Availability

The datasets used and/or analyzed during the current study are deposited at the Department of Social and Clinical Pharmacy, the Faculty of Pharmacy, Charles University, and available from the corresponding author on reasonable request.

## Abbreviations

CAM: Complementary and Alternative Medicine
EAACI: The European Academy of Allergy and Clinical Immunology
NCCIH: The National Centre for Complementary and Integrative Health
TCM: Traditional Chinese Medicine
WAO: The World Allergy Organization
